# Potentiation of Neuronal Nicotinic Receptors by 17β-Estradiol: Roles of the Carboxy-Terminal and the Amino-Terminal Extracellular Domains

**DOI:** 10.1371/journal.pone.0144631

**Published:** 2015-12-18

**Authors:** Xiaochun Jin, Joe Henry Steinbach

**Affiliations:** 1 Department of Anesthesiology, Washington University School of Medicine, Saint Louis, Missouri, United States of America; 2 The Taylor Family Institute for Innovative Psychiatric Research, Washington University School of Medicine, Saint Louis, Missouri, United States of America; Creighton University, UNITED STATES

## Abstract

The endogenous steroid 17β-estradiol (βEST) potentiates activation of neuronal nicotinic receptors containing α4 subunits. Previous work has shown that the final 4 residues of the α4 subunit are required for potentiation. However, receptors containing the α2 subunit are not potentiated although it has these 4 residues, and only one amino acid difference in the C-terminal tail (FLAGMI vs. WLAGMI). Previous work had indicated that the tryptophan residue was involved in binding an analog of βEST, but not in potentiation by βEST. To determine the structural basis for the loss of potentiation we analyzed data from chimeric subunits, which indicated that the major factor underlying the difference between α2 and α4 is the tryptophan/phenylalanine difference, while the N-terminal extracellular domain is a less significant factor. When the tryptophan in α4 was mutated, both phenylalanine and tyrosine conferred lower potentiation while lysine and leucine did not. The reduction reflected a reduced maximal magnitude of potentiation, indicating that the tryptophan is involved in transduction of steroid effects. The regions of the α4 N-terminal extracellular domain involved in potentiation lie near the agonist-binding pocket, rather than close to the membrane or the C-terminal tail, and appear to be involved in transduction rather than binding. These observations indicate that the C-terminal region is involved in both steroid binding (AGMI residues) and transduction (W). The role of the N-terminus appears to be independent of the C-terminal tryptophan and likely reflects an influence on conformational changes caused during channel activation by agonist and potentiation by estradiol.

## Introduction

Heteromeric neuronal nicotinic acetylcholine receptors are pentamers of homologous subunits, containing both α (α2-α6) and β (β2-β4) subunits. In the peripheral nervous system they underlie fast synaptic transmission, while in the central nervous system their major physiological role is to modulate the actions of other transmitters [1, 2]. Genetic studies in humans and molecular manipulations in animals have indicated an important role for heteromeric nicotinic receptors in a variety of diseases or behaviors, including attention, depression, epilepsy, pain perception, and nicotine dependence [2–8]. The most common subunits in the brain are the α4 and β2 subunits, but heteropentameric receptors may contain one or more additional subunits [2, 3].

The endogenous steroid 17β-estradiol (βEST) potentiates neuronal nicotinic receptors containing the α4 subunit [9–11]. Previous studies have demonstrated that the C-terminal sequence of the α4 subunit is essential for potentiation [9–11]. The final 4 residues (alanine-glycine-methionine-isoleucine; AGMI) are required, while the tryptophan preceding them (WLAGMI) appears to be involved in binding an analogue of βEST [11]. Indeed, transferring these 6 amino acids to the C-terminus of the β2 subunit was sufficient to confer potentiation on a receptor when the α4 subunit was mutated to ablate potentiation [10]. Therefore it was surprising when we found that the nicotinic α2 subunit was unable to support potentiation even though its final residues were identical to that of α4 except for the replacement of tryptophan with phenylalanine (FLAGMI). This observation demonstrated that additional structural features are more critical than previously proposed.

To determine the structural basis for the difference between the α2 and α4 subunits, we generated chimeric α subunits and found that the major determinants of the difference lay in the tryptophan-phenylalanine difference and in the nature of the N-terminal extracellular domain. The tryptophan was mutated to 8 other amino acids; other aromatic amino acids (phenylalanine, tyrosine) reduced potentiation while leucine or lysine did not. Analysis of the concentration-potentiation relationship for βEST indicated that the tryptophan is involved in transduction of binding to potentiation. Mutations to a region in the N-terminal domain of the α4 subunit could remove potentiation; this region is near the transmitter-binding site, far from the membrane or the C-terminal tail. Overall the results demonstrate a unique role for the α4 subunit in conferring potentiation by 17β-estradiol. Furthermore, they indicate that the C-terminal region is involved in both binding of βEST and transduction of binding to potentiation, while the N-terminal domain can play an independent role in enhancing the efficacy of βEST.

## Materials and Methods

### Ethics statement

*Xenopus* oocytes were prepared in Dr. C. Zorumski's laboratory (Washington University, St. Louis MO). The protocols for animal use have been approved by the Washington University Animal Studies Committee. Animals are purchased from approved suppliers and are cared for in a Washington University Animal Care Facility. This facility is operated under the supervision of the Washington University Division of Comparative Medicine. This is an approved animal care facility providing housing and veterinary care for animals. Frogs were anaesthetized with 0.1% tricane (3-aminobenzoic acid ethyl ester) during a partial ovariectomy. Eggs are harvested from frogs twice per frog. Frogs are killed under tricaine anesthesia by rapid decapitation. This is an approved method of euthanasia.

### Generation and expression of constructs

The methods used were essentially as described earlier [10, 11]. In brief, constructs of the coding region of the human α4 and β2 subunits were obtained from Dr. Jon Lindstrom (University of Pennsylvania). Constructs of the mouse α2 and β4 subunits were kindly provided by Dr. Jim Boulter (UCLA). The mouse subunits were supplied in pSGEM. All other subunits were in the pCDNA3 vector (Invitrogen, Carlsbad CA). Chimeric subunits were constructed by overlap extension and smaller mutations were constructed using QuikChange (Stratagene, La Jolla CA), and the complete coding region was sequenced to determine that only the desired changes had been made. cRNA was synthesized using the mMessage mMachine T7 kit (Ambion, Austin TX). The concentration of mRNA was estimated from the OD_260_ value. Subunits were injected at a ratio of 8:1 (α:β) for combinations of an α subunit with β2 and 20:1 with β4, to ensure a subunit ratio of 3α:2β. We tested responses of receptors containing either the human or mouse form of the β2 subunit and found no differences (data not shown) and so the data are pooled in the Results. Oocytes were injected with 12 to 15 ng of cRNA in a volume of 18 to 23 nL. Oocytes were maintained at 18 C for 2 to 4 days before physiological study.

### Recording and data analysis

Standard methods were used for two-electrode voltage clamp of *Xenopus* oocytes [11, 12], using an OC-725C voltage clamp (Warner Instruments, Hamden CT). Currents were filtered at 20 Hz, then digitized at 50 Hz (Digidata 1200 interface; Molecular Devices, Sunnyvale, CA) and stored using pClamp 8.0 (Molecular Devices). Transients were analyzed with Clampfit (Molecular Devices). Oocyte recordings were performed in a small chamber (RC-1Z; Warner Instruments Hamden CT) which was continuously perfused with saline. Drug applications were made using a manually controlled perfusion system. The system was made with glass, stainless steel or Teflon components to reduce steroid adsorption. The applications had bath exchange times of ~1 sec. The external solution contained (in mM): 96 NaCl, 2 KCl, 1.8 BaCl_2_, 1 MgCl_2_, and 10 HEPES, pH 7.3. External Ca^2+^ was replaced with Ba^2+^, to avoid activation of Ca^2+^ activated channels. Control experiments showed no difference in effects of 17βEST when the external medium contained Ba^2+^ rather than Ca^2+^ (data not shown).

The concentration-response relationship for activation by ACh was characterized by fitting the Hill equation (Y[ACh] = Ymax (1 / (1 + (EC_50_/[ACh])^nHill), where Y is the response to a concentration of ACh, Ymax is the maximal response, EC_50_ is the concentration producing half-maximal activation, and nHill is the Hill coefficient. Concentration-response data were collected for an individual cell, and data were normalized to the response to 1 mM ACh. The fit was rejected if the estimated error in any fit parameter was greater than 60% of the fit value, and all parameter estimates for that fit were discarded. The relationship was analyzed for each cell, then overall mean values were calculated for oocytes injected with that set of constructs.

Potentiation by 17β-estradiol is strongest for low concentrations of ACh [9, 11]. Since the EC_50_ for activation by ACh depends on the subunit combinations expressed, each oocyte was tested with 1 mM ACh, to estimate the maximal response. A low concentration of ACh, chosen to be able to evoke less than 20% of the maximal current, was then applied. After the response to ACh had reached a stable level, the application was switched to ACh plus 17β-estradiol. After the response to ACh plus βEST had reached the maximal response the application was switched to bathing solution, followed by repeat of the control low [ACh]. The relative response in the presence of 17β-estradiol was then calculated as the ratio of the maximal response to ACh + βEST to the response to ACh alone, immediately preceding the switch to ACh + βEST. ACh or ACh plus 17β-estradiol were applied for 10 to 20 seconds, and applications were separated by 3 to 4 minutes. Because 17β-estradiol is rapidly washed out [9, 11] this allows full recovery. Some oocytes were tested with more than one concentration of ACh or 17β-estradiol. In this case, the combination of concentrations producing the largest potentiation ratio was used as the single value for that particular oocyte. The concentration-response relationship for potentiation was characterized by fitting a 4 parameter Hill equation to the ratios (Z[drug] = 1 + Zmax (1 / (1 + (EC_50_/[drug]^nHill)), where Z is the relative response to a concentration of ACh in the presence of drug, Zmax is the maximal response, EC_50_ is the concentration producing half-maximal potentiation, and nHill is the Hill coefficient. The response to the highest concentration to steroid was sometimes reduced from the maximal, indicating the presence of some inhibition at high concentrations [11, 13]. If the reduction were by more than 10% the response to the highest concentration was omitted from the data for the fit.

The ability of a given receptor to be potentiated was assessed by a one sample test of the potentiation ratio to 1 (no effect). Comparison of potentiation between receptors was assessed by t-test or ANOVA with Bonferroni correction for multiple comparisons and comparison to wild-type receptors by ANOVA with Dunnett’s correction.

Multiple regression analysis of the chimeric constructs was conducted using the source of the sequence in the 4 regions as the independent variables, assigning the number zero (0) to a region containing sequence from the α2 subunit and one (1) to a region containing sequence from the α4 sequence. The regression was performed using individual values (statistics reported in Results) or the mean values. The rank order for P values for the parameters were the same for either regression on individual values or mean values, although the regression on the mean provided much less significant values for P. The coefficients were within 10% of each other for either regression, except for the regression on EC_50_ that also had the smallest number of observations.

The analysis of potentiation for the D to A loop and E loop chimeras (see Results) was performed by arbitrarily assigning a value of 0 to wild-type, 1 when only the D to A loop region was substituted, 2 when only the E loop region was substituted and 3 when both were substituted. A linear regression of potentiation on the structural value was then performed, to indicate whether the effect of the chimeras was different for subunits containing W or F residues.

Values are presented as arithmetic mean ± SE (number of observations). Fits of concentration-response relationships were made using SigmaPlot (Systat Software, Inc., San Jose, CA). Statistical tests were made using Excel (Microsoft, Redmond WA) or STATA (StataCorp LP, College Station, Texas).

The homology model of the human α4 subunit was made by threading the α4 sequence (NCBI Reference Sequence: NP_000735.1) onto the structure for the *C*. *elegans* GluCl channel (3RIF; [14] using the Expasy server (http://swissmodel.expasy.org/). The structure was viewed and the figure prepared using Chimera (http://www.cgl.ucsf.edu/chimera).

### Drugs used

17β-estradiol (βEST; CAS 50-28-2), acetylcholine chloride (ACh; CAS 60-31-1), and 19-norpregna-1,3,5(10)-trien-20-yne-3,17-diol, (17α)-(9CI) (17α-vinyl estradiol; CAS 57-63-6) were purchased from Sigma-Aldrich (St. Louis, MO). Steroids were prepared as a 20 mM stock solution in DMSO and diluted into external solution on the day of an experiment. ACh was prepared as a 1M stock solution in bath solution and stored frozen at -20 C. Working solutions were prepared on the day of experiments.

## Results

### Receptors containing the α2 subunit are not potentiated by 17β-estradiol

**Fig 1:**
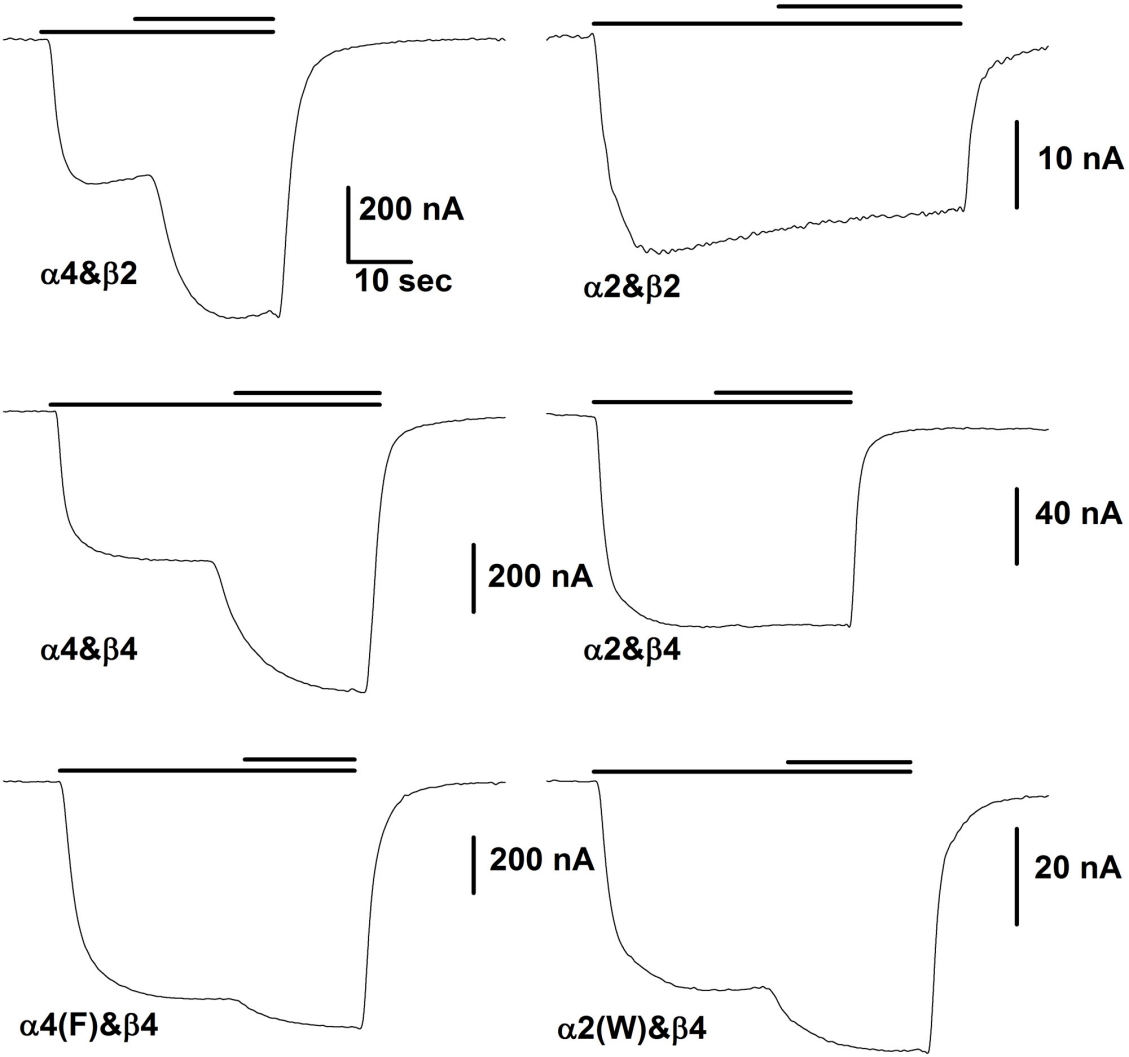
Representative responses of oocytes injected with the indicated subunits. In each case the lower bar above the traces indicates the time of application of ACh while the upper bar shows the time of application of the same concentration of ACh plus 10 μM βEST. The concentration of ACh was chosen to produce a response of about 10% of maximal. The horizontal scale bar in the upper left panel shows 10 sec for all panels. For the trace obtained with α4&β2 the ACh concentration was 3 μM, for α4&β4 3 μM, for α4(F)&β4 3 μM, for α2&β2 10 μM, for α2&β4 20 μM and for α2(W)&β4 20 μM. For the figure the data were smoothed by averaging 10 points.

The initial observation was that 10 μM 17β-estradiol did not potentiate responses from oocytes expressing α2 and β2 subunits (the subunits injected are given as α&β), while it potentiated responses of oocytes expressing α4&β2 receptors (Fig 1). This observation was unexpected, as the α2 subunit has the AGMI sequence at the carboxy-terminal tail.

We found that significantly larger maximal currents (estimated from the response to 1 mM ACh) were expressed in oocytes expressing α2 in combination with the β4 subunit rather than the β2 subunit (α2&β4: 929 ± 102 nA, N = 85; α2&β2 130 ±21, N = 26; mean ± SE, number of observations), as has been reported previously by others [15, 16]. Previous work ([9]; and see below) has shown that receptors containing α4 and either the β2 or β4 subunit are well potentiated by βEST. Because the responses to receptors containing α2 and β2 subunits were so small (Table 1), the majority of the subsequent experiments were performed using the β4 subunit.

**Table 1:** The effect of subunit ratios on properties of the expressed receptors.

Subunits	Potentiation ratio	ACh EC50 (μM)	Maximal response (-nA)
α4&β2 1:8	2.56 ± 0.12 (7) [<0.001; 0.4]	6.6 ± 1.1 (22) [<0.001]	1051 ± 196 (46) [<0.001]
α4&β2 8:1	2.90 ± 0.27 (20) [<0.001; -]	132 ± 10 (36)	12971 ± 1256 (53)
α4&β4 1:10	1.48 ± 0.07 (13) [<0.001; <0.001]	14 ± 2.4 (21) [<0.001]	14898 ± 1221 (27) [0.002]
α4&β4 20:1	1.87 ± 0.04 (80) [<0.001; -]	31 ± 1.9 (21)	10607 ± 473 (118)
α2&β2 1:8	1.25 ± 0.05 (16) [[<0.001; <0.001]	3.9 ± 0.3 (37) [<0.001]	190 ± 35 (59) [0.14]
α2&β2 8:1	0.96 ± 0.04 (12) [0.36; -]	217 ± 42 (15)	130 ± 21 (26)
α2&β4 1:8	1.01 ± 0.01 (11) [0.45; 0.8]	22 ± 3 (15) [<0.001]	11509 ± 1635 (22) [<0.001]
α2&β4 20:1	1.01 ± 0.02 (30) [0.37; -]	91 ± 6 (28)	929 ± 102 (85)

The first column gives the subunits and ratio of cRNA injected. The second column gives potentiation ratio for βEST, as the mean ± SE (number of observations), with two probabilities in the square brackets. The first is the probability that the potentiation ratio is actually 1 (no effect; one sample t-test) and the second is the probability that the potentiation for the two injection ratios are the same (unpaired t-test). The second and third columns give similar data for the EC_50_ for ACh and the maximal response; for these a single probability is given for the probability that the values are the same for the two injection ratios.

We tested the consequences of expressing subunits at different ratios, with the results summarized in Table 1. As can be seen, the different injection ratios resulted in receptors that differed in terms of the concentration of ACh producing a half-maximal response (EC_50_), indicating that the ratios used resulted in receptors with biased representation of the expected stoichiometries [10, 17, 18]. In terms of potentiation by βEST, receptors containing α4 subunits were consistently potentiated and the larger ratio of α4 to β subunits resulted in larger potentiation. Previous work has shown that potentiation increases with the number of α4 subunits [10]. Receptors containing α2 subunits were not significantly potentiated, except for α2&β2 at a 1:8 ratio that was weakly potentiated. In all subsequent studies we used an excess of α subunit (20:1 with the β4 subunit and 8:1 with the β2 subunit) to result in receptors containing a 3:2 α:β subunit ratio.

Previous work had indicated the C-terminal region of the receptor is critical for potentiation by βEST. The final transmembrane-spanning helix (TM4) in the α4 subunit is followed by a short C-terminal domain with the sequence PPWLAGMI. That work found that mutations to the final 4 residues in the α4 subunit significantly reduced or completely removed potentiation [11]. Mutation of the leucine to alanine, and mutation of the tryptophan to leucine or alanine did not affect potentiation by βEST. However, we found that an analogue of βEST, 17α-vinyl estradiol, was able to potentiate receptors containing an α4 subunit that had been mutated to remove potentiation by βEST, and that mutation of W to L removed this residual potentiation. We concluded that the tryptophan interacted with the π-electrons of the vinyl substituent and allowed binding of the analogue to the receptor.

The α2 subunit has the critical 4 residues at the C-terminal (α4: WLAGMI; α2: FLAGMI) so we were surprised that it did not support potentiation by βEST. To explore the reason for the lack of potentiation, we first mutated the α4 subunit to change the tryptophan to phenylalanine (α4(F)) and made the complementary mutation in α2 (α2(W)). Potentiation of α4(F) was significantly reduced (α4&β4 1.85 ± 0.03, N = 129 vs. α4(F)&β4 1.26 ± 0.03, N = 81; P < 0.001 by ANOVA with Bonferroni correction for this group of 4 receptors), while potentiation was conferred on α2(W) (α2&β4 1.02 ± 0.02, N = 28 vs. α2(W)&β4 1.31 ± 0.03, N = 31; P < 0.001). However, potentiation of α2(W) was significantly less that of α4 (P < 0.001) and potentiation of α4(F) was significantly greater than α2 (P = 0.002). These observations indicated that the change from W to F is a major contributor to the loss of the ability of βEST to potentiate responses, but additional elements in the subunit also are relevant.

**Fig 2:**
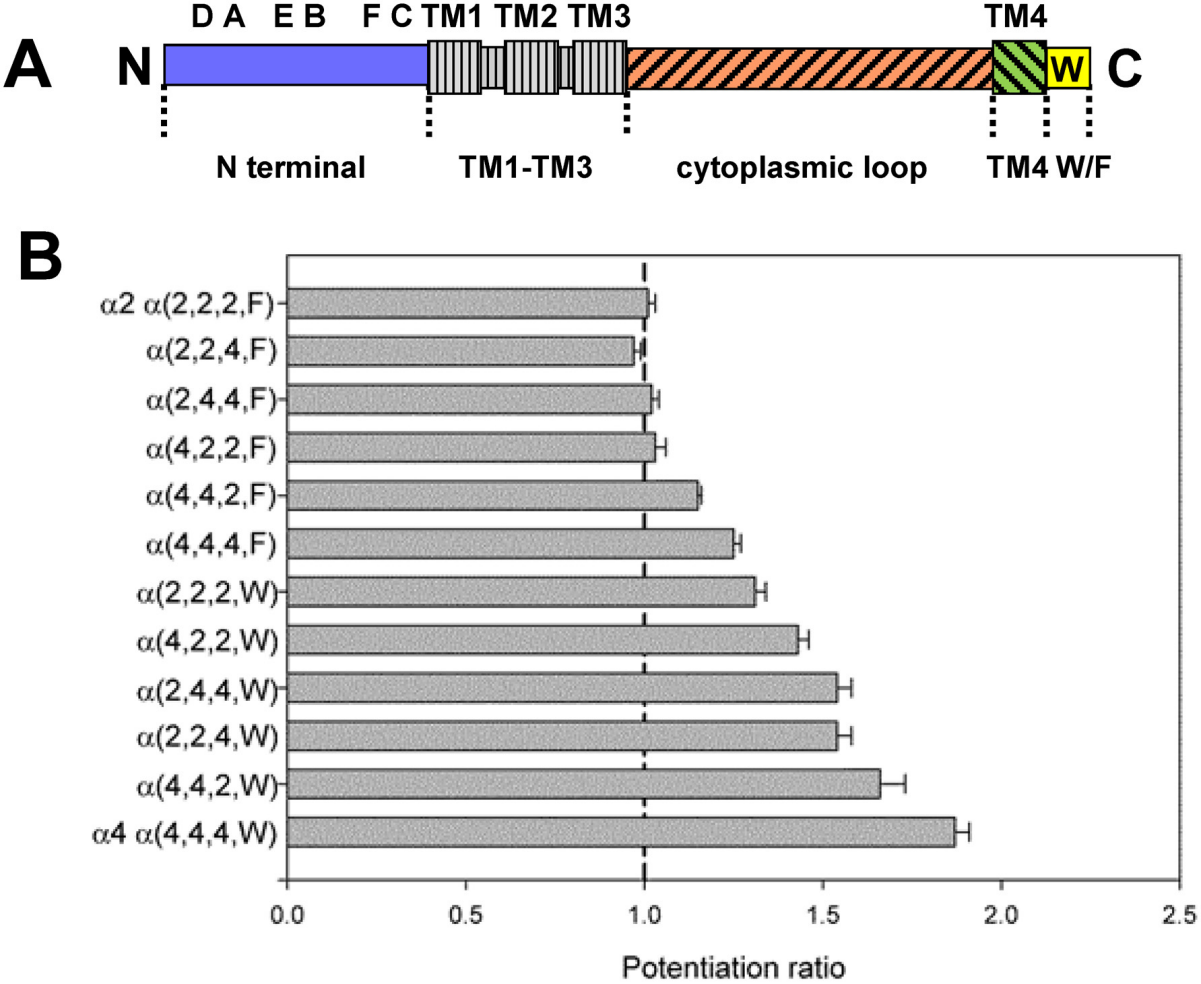
Chimeric subunits used to determine regions contributing to potentiation by βEST. Panel A shows a linear cartoon of a nicotinic subunit, with the amino-terminus (“N”) at the left. Some major structural features are indicated: in the amino-terminal extracellular domain there are 6 domains involved in agonist binding, the A, B, C loops contributed by the α subunit and the D, E, F loops contributed by the β subunit. The 4 transmembrane regions are indicated by the enlarged boxes; the channel is formed by TM2 helices contributed by the 5 subunits in the receptor. The 4 regions studied were the N-terminal domain (blue; extending from the amino-terminus to V(210)IRRLP sequence at the start of TM1 (the amino acid residue numbering is for the mature α4 subunit; NCBI Reference Sequence: NP_000735.1). The TM1-TM3 regions (gray with vertical hatching) are identical in the mouse α2 and human α4 subunits. The region called the cytoplasmic loop (red with forward hatching) extends from N(301)VHHRSP at the end of TM3 to D(571)RIFL at the start of TM4. TM4 (green with backwards hatching) is the region from D(571)RIFL to FLP(592)P. The tryptophan in α4 is in the carboxy-terminal region, W(594)LAGMI. The cytoplasmic loop is highly divergent in nicotinic subunits, and contains between about 150 and 270 amino acids. Panel B shows the mean potentiation ratio for the chimeras studies. Chimeras are identified by which subunit contributed the relevant portion of the construct, so α(2,2,4,W) indicates an α subunit with α2 sequence in the amino-terminus, α2 sequence in the cytoplasmic loop, α4 sequence in the TM4 region and W in the C-terminal tail. The figure shows the mean ± SE potentiation ratio produced by βEST (see Table 2 for numbers of observations).

To determine which regions made major contributions we constructed a number of chimeric subunits between the α2 and α4 subunit and assessed the ability of βEST to potentiate responses. The constructs were made to exchange sequence in 4 regions of the subunit as shown in Fig 2: the N-terminal extracellular domain, the large intracellular loop between the TM3 and TM4 domains, the TM4 domain and the W/F difference in the C-terminal tail. We note that the first 3 membrane spanning regions, that include the major channel-lining portions of the subunit, are identical between α2 and α4. For simplicity the constructs are abbreviated as α(subunit contributing the region), so α(4.4.4.W) indicates the α4 wild-type subunit and α(2.2.2.F) the α2 subunit. A first inspection of Fig 2 and Table 2 indicates that the presence of a tryptophan, rather than phenylalanine, is a very strong determinant. We then performed multiple linear regression (see Methods) of the extent of potentiation on the source of sequence in the various regions, that confirmed that the most significant factor was the presence of W rather than F (P < 10^−30^ that the coefficient differs from zero). The second most important factor was the presence of α4 sequence in the N-terminal extracellular domain (P = 4x10^-9^) with lesser contributions from the M4 domain (P = 6x10^-5^) and the least from the M3-M4 main cytoplasmic loop (P = 0.006) (Table 2). In each case the sign of the dependence indicated that presence of sequence for the α4 subunit was associated with larger potentiation. Accordingly we first focused our analysis on the tryptophan residue.

**Table 2:** A summary of the data used in the multiple regression to identify important regions in the α subunit.

Construct	N	cyto	TM4	W/F	Potentiation ratio	ACh EC_50_ (μM)	Maximal response (-nA)
**α2** α(2.2.2.F)	2	2	2	F	1.01 ± 0.02 (30) [0.4]	91 ± 6 (28)	929 ± 102 (85)
α(2.2.4.F)	2	2	4	F	0.97 ± 0.02 (16) [0.16]	ND	2526 ± 567 (17)
α(2.4.4.F)	2	4	4	F	1.02 ± 0.02 (8) [0.4]	ND	3367 ± 627 (20)
α(4.2.2.F)	4	2	2	F	1.03 ± 0.03 (24) [0.3]	63 ± 5 (8)	875 ± 180 (30)
α(4.4.2.F)	4	4	2	F	1.15 ± 0.01 (20) [<0.001]	46 ± 10 (3)	7827 ± 615 (28)
α(4.4.4.F)	4	4	4	F	1.25 ± 0.02 (35) [<0.001]	32 ± 3 (3)	9146 ± 505 (42)
α(2.2.2.W)	2	2	2	W	1.31 ± 0.03 (31) [<0.001]	103 ± 3 (9)	751 ± 66 (38)
α(4.2.2.W)	4	2	2	W	1.43 ± 0.03 (14) [<0.001]	ND	2083 ± 314 (12)
α(2.4.4.W)	2	4	4	W	1.54 ± 0.04 (23) [<0.001]	76 ± 3 (3)	6881 ± 602 (23)
α(2.2.4.W)	2	2	4	W	1.54 ± 0.04 (10) [<0.001]	99 ± 6 (6)	651 ± 85 (20)
α(4.4.2.W)	4	4	2	W	1.66 ± 0.07 (12) [<0.001]	41 ± 5 (2)	9039 ± 673 (12)
**α4** α(4.4.4.W)	4	4	4	W	1.87 ± 0.04 (80) [<0.001]	31 ± 2 (21)	10607 ± 473 (118)

The first column gives the name for the construct. All α constructs were expressed with the β4 subunit at a 20:1 ratio. The next 4 columns show the structure of the chimeras used, where N indicates the amino-terminal extracellular domain, cyto indicates the major cytoplasmic loop, TM4 indicates the fourth transmembrane helix and W/F indicates the nature of the residue in the C-terminal tail. The regions are indicated schematically in Fig 2A and the residue locations for the joining points are given in the legend to Fig 2. Data for α2 is shown in the first line, and for α4 in the last, while the other constructs are arranged in order of increasing potentiation. Note that potentiation segregates completely on the basis of the W/F difference, and within either W or F generally increases when the N-terminal domain is contributed by α4. The final two columns give data on the concentration of ACh producing a half-maximal response (ACh EC_50_) and the response to a high concentration of ACh (Maximal response). The data are shown as mean ± SE (N), and the number in square brackets gives the probability that the potentiation ratio is equal to 1 (no potentiation). ND: not determined.

### Properties of the tryptophan residue

It was surprising that mutation of tryptophan to phenylalanine significantly reduced potentiation, because in previous work we had examined effects of mutations of W to L and A, and had shown that these mutations did not affect potentiation [11]. To examine the dependence on amino acid structure we mutated the tryptophan in the α4 subunit to a number of other amino acids (D, F, G, K, S and Y) as well as A and L. The consequences of these mutations on potentiation are shown in Table 3. Unexpectedly, changes to other aromatic residues (tyrosine, phenylalanine) greatly reduce potentiation, while mutations to leucine, alanine, lysine or serine have no significant effect. To determine whether the effects on potentiation were correlated with a physical property we performed regressions of potentiation on several properties of amino acids. Regressions were performed using both parameter values and the rank of the parameter, to avoid effects of outliers. We used properties of the side chain: volume [19], surface area [20], bulk [21], hydropathy [21, 22], and polarizability [23]. We also examined some properties of amino acids in terms of protein structure: propensity to occur in α-helices or β sheets [24] or at the end of membrane spanning helices [25]. Finally, to assess a possible role in anchoring a transmembrane helix we examined an interfacial hydrophobicity scale [26]. In no case was the correlation significantly different from zero. We also examined correlations with two measures of amino acid similarity based on replacement in related sequences, the BLOSUM62 index [27] and the PAM250 index [28]. Again, there was no significant correlation. The lack of any significant correlations is unexpected, and hence the critical property of the amino acid at this position remains unknown.

**Table 3:** The effects of mutations of the tryptophan residue (PPWLAGMI).

Construct	Potentiation ratio	ACh EC_50_ (μM)	Maximal response (-nA)
α4(D)	1.04 ± 0.01 (12) [0.002; <0.0001]	22 ± 2 (5)	10398 ± 1333 (13)
α4(F)	1.25 ± 0.02 (35) [<0.001; <0.0001]	32 ± 3 (3)	9146 ± 505 (42)
α4(Y)	1.33 ± 0.03 (12) [<0.001; <0.0001]	ND	2394 ± 382 (12)
α4(G)	1.46 ± 0.04 (15) [<0.001; <0.0001]	ND	3001 ± 503 (17)
α4(L)	1.77 ± 0.18 (7) [<0.001; 0.95]	ND	7418 ± 632 (7)
α4(W) wt	1.87 ± 0.04 (80) [<0.001; —]	31 ± 2 (21)	10607 ± 473 (118)
α4(A)	1.88 ± 0.03 (4) [<0.001; 1.0]	ND	7390 ± 719 (4)
α4(S)	1.90 ± 0.05 (11) [<0.001; 0.9]	21 ± 2 (6)	10964 ± 927 (11)
α4(K)	1.97 ± 0.14 (10) [<0.001; 0.8]	14 ± 2 (7)	12244 ± 1392 (11)

The first column gives the α4 subunit (expressed with β4 at a 20:1 α:β ratio). The second column gives the potentiation ratio produced by βEST (mean ± SE (N)). In square brackets are the probability that the potentiation ratio is equal to 1, followed by the probability the potentiation ratio is equal to that of wild-type (α4(W)), ANOVA with Dunnett's correction). The third column gives the EC_50_ for ACh activation and the last column the maximal response.

Previous work had found that the spatial position of the AGMI residues is critical. Potentiation was lost when single residues were inserted or deleted from the sequence between the di-proline sequence at the proposed end of the TM4 region and GMI [11]. Accordingly we examined whether the position of the W is also critical. These constructs were made using the α4(F) subunit as a base, to determine whether W inserted in other positions could substitute for W in the wild-type position (Table 4). We found that when W was moved to other positions in the C-terminal tail it did not result in potentiation greater than α4(F), indicating that the spatial position of the tryptophan side chain is important. α4(WPFLAGMI) and α4(PPFWAGMI) showed indistinguishable potentiation from α4(F), suggesting that the position of GMI motif has not been significantly altered. However the α4(PWFLAGMI) construct resulted in significantly less potentiation, perhaps because substitution of that proline affected the position of the GMI motif. Previous work had found that replacement of both prolines with glycine removed potentiation [11], while replacement of the first proline with glutamine (PP to QP) had no effect [10].

**Table 4:** The effects of moving the tryptophan to neighboring positions.

Construct	Potentiation ratio	ACh EC_50_ (μM)	Maximal response (-nA)
α4(PPFLAGMI)	1.25 ± 0.02 (35) [<0.001; --]	32 ± 3 (3) [--]	9146 ± 505 (42)
α4(WPFLAGMI)	1.32 ± 0.06 (5) [0.005; 0.5]	7 ± 2 (4) [<0.001]	11522 ± 1941 (5)
α4(PWFLAGMI)	1.05 ± 0.05 (8) [0.3; <0.001]	7 ± 2 (8) [<0.001]	15190 ± 1725 (10)
α4(PPFWAGMI)	1.36 ± 0.06 (6) [0.001; 0.12]	14 ± 1 (4) [0.006]	12886 ± 1447 (8)

The first column gives the subunit (expressed with β4 at a 20:1 α:β ratio). The second column gives the potentiation ratio produced by βEST (mean ± SE (N)). In square brackets are the probability that the potentiation ratio is equal to 1, followed by the probability the potentiation ratio is equal to that of α4 with W mutated to F (α4(PPFLAGMI), ANOVA with Dunnett's correction). This comparison is made to determine whether any of the constructs with an introduced W potentiates significantly better than the W to F mutation. The third column gives the EC_50_ for ACh activation with the probability the EC_50_ is equal to that of α4(PPFLAGMI) and the last column the maximal response.

**Fig 3:**
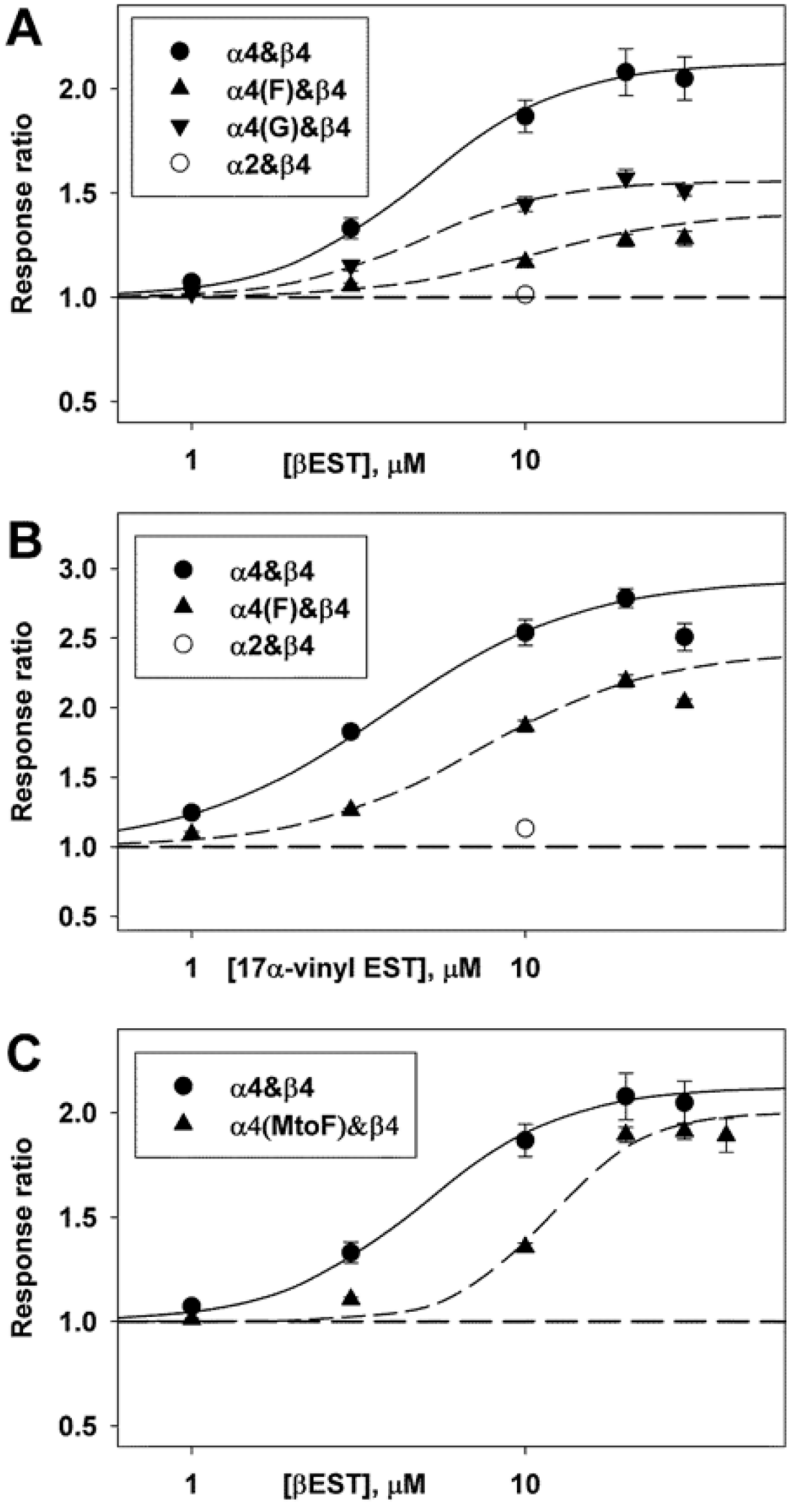
The effects of mutations on the concentration-effect relationship for potentiation. Data are shown for mutations of the tryptophan residue (**W**LAGMI; panels A and B) and the methionine residue (WLAG**M**I; panel C). ACh was applied at a concentration producing a response that produced a response of about 5 to 7% of maximal in the absence of steroid. The α subunit was expressed with β4 at an injection ratio of 20:1. The concentration-response relationship for potentiation was characterized by fitting a 3 parameter Hill equation to the ratios (Z[drug] = 1 + Zmax (1 / (1 + (EC_50_/[drug])^nHill), where Z is the response to a concentration of ACh in the presence of drug relative to that in the absence, Zmax is the maximal effect, EC_50_ is the concentration producing half-maximal potentiation, and nHill is the Hill coefficient. The fits did not include points that appeared to demonstrate block (e.g. data with 30 μM 17α-vinylestradiol). The data from each cell were fit separately, giving the following mean parameter estimates for βEST: α4: Zmax = 1.12 ± 0.12 (11 cells) and EC_50_ = 4.92 ± 0.48 μM; for α4(F): 0.41 ± 0.06 (3) P = 0.0002 and 10.43 ± 2.00 μM P = 0.4; for α4(G): 0.56 ± 0.03 (4) P = 0.001 and 4.90 ± 0.38 μM P = 0.98; and for α4(MtoF): 1.00 ± 0.03 (8) P = 0.34 and 11.76 ± 0.39 μM P < 0.0001, with values given as mean ± SE (number of cells) and significance of difference to α4 by t-test. The best-fitting estimates for 17α-vinylestradiol are: α4: 1.93 ± 0.06 (3) and 3.84 ± 0.27 μM and α4(F): 1.41 ± 0.08 (4) P = 0.004 and 7.32 ± 0.68 μM P = 0.010. The points show mean ± SE while the lines show the relationship predicted by the mean fit parameters.

Finally, we examined the mechanistic basis for the reduced potentiation produced by some mutations of the tryptophan. Is there a change in affinity for βEST or a change in maximal efficacy for potentiation? Potentiation at a single concentration of drug cannot distinguish these alternatives. Accordingly, we tested selected subunit combinations with various concentrations of βEST. We also examined 17α-vinyl estradiol. Our previous work had concluded that this analogue interacted specifically with the tryptophan residue [11] and so this appeared to be a valuable test. The results are shown in Fig 3, and indicate that the major effect is to reduce the maximal extent of potentiation, with a smaller effect on the concentration producing half-maximal potentiation. We note that with these steroids the aqueous concentration may be limited by their low solubility. It is also the case that βEST and other steroids inhibit these receptors at higher concentrations [11, 13] and that potentiation and inhibition are mediated by different regions of the receptor [11]. For these reasons the responses to higher concentrations may be reduced and so these observations must be taken with some circumspection. However, they indicate that the major role of the residue at the position occupied by tryptophan is to transduce binding to potentiation.

For comparison we determined the effects of a mutation in the GMI motif, mutating methionine to phenylalanine. This mutation was chosen because previous work [11] had indicated that potentiation by 10 μM βEST was reduced but not eliminated, which indicated that any shift in concentration dependence would be observable in the accessible aqueous concentration range of βEST. As shown in Fig 3C this mutation shifted the concentration response curve to higher concentration but did not reduce the maximal potentiation significantly, indicating that this residue is involved in steroid binding.

### Relationship between potentiation and activation by ACh

We performed regressions of potentiation on EC_50_ and maximal response, using data obtained when the β4 subunit was expressed with an α subunit. For regression of potentiation on EC_50_ the coefficient was not significantly different from zero (P = 0.28; 31 subunit combinations examined). For regression on maximal response the correlation coefficient differed from zero (P = 0.02; 43 subunit combinations) (the constructs studied are listed in S1 Table). Further examination of the data indicated that the correlation likely arose from the fact that receptors containing more α4 sequence had both larger potentiation and larger maximal response.

An independent indication that potentiation is not associated with activation is given by analysis of the chimeric receptors (Table 2). Multiple regression analysis was performed in the same manner as for the extent of potentiation, but in this case using the EC_50_ value for ACh and the maximal elicited current as dependent variables. Regression of the ACh EC_50_ values (9 subunit combinations; Table 2) indicated that the most important factor was the presence of α4 sequence in the amino-terminus (P = 6x10^-6^) with the cytoplasmic (TM3-TM4) loop less important (P = 0.005), while other coefficients were not significantly different from zero. The sign of the coefficients indicated that presence of α4 sequence was associated with a smaller value for the EC_50_. A similar analysis of the maximal current values (12 subunit combinations; Table 2) indicated that the cytoplasmic loop was the major factor (P = 7x10^-23^) followed by the amino-terminal region (P = 2x10^-9^), the W/F difference (P = 9x10^-3^) and the TM4 region (P = 0.03). The sign of the coefficients indicated that presence of α4 sequence was associated with a larger maximal response. Two conclusions are suggested by these observations. First, potentiation, maximal response and ACh EC_50_ appear to be most strongly influenced by different regions of the subunit and so are likely to be independent, at least for these subunit combinations. Second, the results are largely consistent with other work indicating that the amino-terminal domain (containing the agonist-binding site) is critical for agonist binding and receptor activation which would be reflected in the EC_50_ value for ACh. Receptor trafficking is strongly influenced by the cytoplasmic loop and assembly by the amino-terminal domain, both of which would be reflected in the different maximal response values.

### Properties of the N-terminal extracellular domain

The region with the second most significant factor in the multiple regression analysis was the amino-terminal domain. Our previous work had suggested that the N-terminal domain played little if any role in potentiation, because we were able to ablate potentiation by mutations to the C-terminus of the α4 subunit. We could then fully restore potentiation by transferring the WLAGMI sequence to the C-terminus of the β2 subunit [10]. Since the α4 and β2 subunits are divergent in the amino-terminus extracellular domain (50% identity) it had seemed unlikely that the nature of the amino-terminal region of the subunit containing the C-terminal sequence contributed to potentiation. Accordingly it was surprising to us that the present studies of the α2 and α4 subunit indicated an appreciable role.

There seem to be two physically distinct possibilities for the involvement of the N-terminal domain in potentiation. One involves a relatively close interaction between some portion of the N-terminal domain and the C-terminal domain, for instance a contribution to a binding site for βEST or an interaction between amino acid side-chains in the two domains that are required for coupling steroid binding to gating. The second possibility does not require a close contact but instead relies on a contribution of the N-terminal to the gating or transduction processes. To probe these possibilities we examined potentiation of receptors containing a number of chimeric subunits.

**Fig 4:**
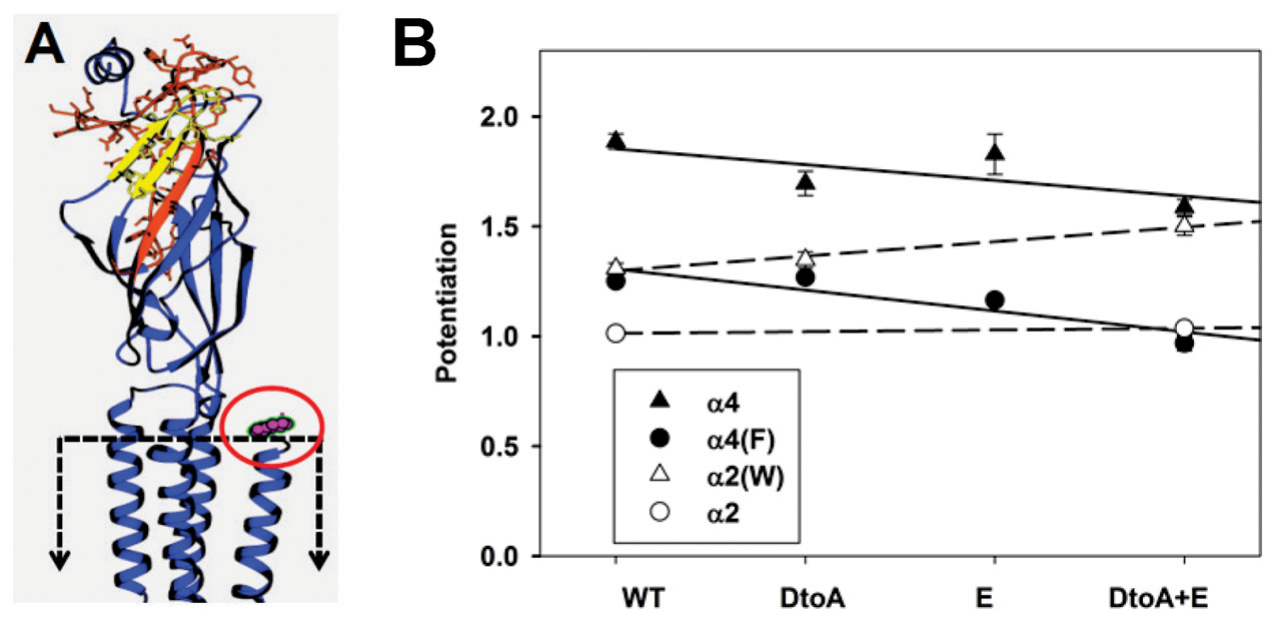
The effects of N-terminal chimeras on potentiation. Panel A shows a homology model of the α4 subunit (see Methods). A single subunit is shown, with the ion channel to the left and the extracellular solution at the top. The approximate location of the membrane is shown by the dashed box, note that the membrane spanning region is truncated in this view. The D to A loop region (MMT(57) to IVL(97)) is shown in red with amino acid side chains shown in stick representation, while the E loop (VTH(109) to QWT(124)) is shown in yellow. The carboxyl terminal region is shown in magenta and indicated by the red oval. In aligning the α4 subunit to sequence in the GluCl structure, the last amino acid in the structure aligned with the leucine in α4, so the AGMI residues are not shown. Note that the regions exchanged are far from the membrane and the C-terminal domain, but include portions of the ACh-binding site. Panel B shows potentiation for the different chimeras. The chimeric regions interchanged are indicated along the abscissa; chimeras were constructed on the α4 (filled triangles) and α4(F) (filled circles) subunits inserting sequence from the α2 subunit. Homologous transfers were made by transferring sequence from the α4 subunit into the α2 (hollow circles) and α2(W) (hollow triangles) subunits. The lines through the points for points show the lines predicted by linear regression on the source of the inserted region (see Methods). The slopes are -0.072 for chimeras on α4 and -0.095 on α4(F), and 0.008 for α2 and 0.066 for α2(W). None of the slopes differ significantly from 0 (P > 0.09). However, as shown in Table 5 in all cases the potentiation of receptors containing α4(DtoA+E) regions was significantly larger than those containing the homologous α subunits containing α2(DtoA+E) regions. Data values are given in Table 5.

We tried to remove potentiation from the α4 subunit by inserting regions of the α2 subunit. Our initial coarse chimeric constructs showed that the construct α(2.4.4.W) showed reduced potentiation compared to α(4,4,4,W) and α(2.4.4.F) had no potentiation (Table 2). We used finer chimeras of the N-terminal domain to localize the critical regions. Chimeras were constructed on both the α4 and α4(F) subunit to examine whether there was an interaction between the N-terminal region and the tryptophan. Preliminary results (data not shown) suggested that regions near the ACh-binding site might be important, and further work indicated that the regions covering the D loop to the A loop, and separately the E loop reduced potentiation (amino acid residues are given in the legend to Fig 4). Substitution of both the D to A and E Loops further reduced potentiation when made on the α4 subunit, and completely removed it when made on the α4(F) subunit (Fig 4B and Table 5). The contributions of the W/F change and the mutations in the N-terminal region appear to be independent, as the data for the α4 and α4(F) subunits fall on two parallel lines (Fig 4B). When we made reciprocal constructs, swapping in the α4 D to A and E Loop regions into the α2 subunit there was only a small potentiation (Table 5). When the α2(W) subunit was used there was a more marked increase in potentiation, and the slope was close to the inverse of the slope when α2 sequence was placed into the α4 subunit (Fig 4B). Although it was possible to increase potentiation in the α2 subunit by inserting these regions form the α4 subunit, there appear to be additional regions that affect potentiation, since in most cases reciprocal constructs based on the α2 subunit resulted in less potentiation than those based on α4 (e.g. α4 versus α2(W)(α4DtoA+E) or α4(F) versus α2(α4DtoA+E); Table 5).

**Table 5:** The effects of N-terminal chimeras on potentiation.

Construct	Potentiation ratio	ACh EC_50_ (μM)	Maximal response (-nA)
α4	1.87 ± 0.04 (80) [<.001, -]	31 ± 2 (21)	10607 ± 473 (118)
α4(α2DtoA)	1.70 ± 0.06 (4) [0.001, 0..5]	76 ± 12 (4)	12160 ± 390 (11)
α4(α2E)	1.83 ± 0.09 (14) [<.001, 0.9]	6.9 ± 0.5 (4)	7765 ± 366 (4)
α4(α2DtoA+E)	1.59 ± 0.04 (5) [<.001, 0.12]	ND	13221 ± 926 (12)
α(F)	1.25 ± 0.02 (35) [<.001, -]	32 ± 3 (3)	9146 ± 505 (42)
α4(F)(α2DtoA)	1.27 ± 0.01 (13) [<.001, 0.9]	73 ± 4 (4)	13760 ± 1143 (13)
α4(F)(α2Eloop)	1.16 ± 0.02 (15) [<.001, 0.015]	8.5 ± 0.6 (11)	7173 ± 705 (15)
α4(F)(α2DtoA+E)	0.97 ± 0.03 (13) [0.4, <.001]	16 ± 2 (6)	12361 ± 1017 (14)
α2	1.01 ± 0.02 (30) [0.5, -]	91 ± 6 (28)	929 ± 102 (85)
α2(α4DtoA+E)	1.04 ± 0.01 (13) [0.012, 0.4]	257 ± 12 (23)	544 ± 42 (39)
α2(W)	1.31 ± 0.03 (31) [<.001, -]	103 ± 3 (9)	751 ± 66 (38)
α2(W)(α4DtoA)	1.35 ± 0.04 (15) [<.001, 0.6]	77 ± 7 (7)	211 ± 30 (15)
α2(W)(α4DtoA+E)	1.50± 0.04 (17) [<.001, <.001]	318± 30 (7)	145± 16 (17)

The first column gives the α subunit (expressed with β4 at a 20:1 α:β ratio). The constructs used are given in the Legend to Fig 4. The second column gives the potentiation ratio produced by βEST (mean ± SE (N)). In square brackets are the probability that the potentiation ratio is equal to 1, followed by the probability the potentiation ratio is equal to that of the first subunit is the group, ANOVA with Dunnett's correction). The third column gives the EC_50_ for ACh activation and the last column the maximal response.

The D to A and E Loop regions are located far from the membrane in a homology model based on the GluCl structure (Fig 4A, [14]) and so the results argue against the first possibility, that requires a close physical association between the C-terminal tail and specific domains in the amino-terminal extracellular region.

We then examined the possibility that the contribution of the α N-terminal region to potentiation occurred even when the C-terminal region was located on a different subunit. To do this we utilized a β2 subunit construct in which we had incorporated the carboxy-terminal domain conferring potentiation by βEST—β2(WLAGMI) [10]. We compared the amount of potentiation seen when this chimeric subunit was expressed with several α constructs to that seen when the wild-type β2 subunit was expressed. For α subunits we chose constructs that were identical in the TM4 region and at the W/F site but differed in the amino-terminal domain. Note that the additional βEST binding and transduction domains were located on a separate subunit from the α subunit amino-terminal domain. The results are shown in Table 6. Comparing α(4.4.4.W) to α(2.2.4.W) potentiation was significantly larger when α(4.4.4.W) was expressed with either β2 (P = 5 x 10^−5^ 2-tailed t-test) or β2(WLAGMI) (P = 4 x 10^−3^). Similarly, comparing α(4.4.2.F) to α(2.2.2.F) the potentiation was significantly larger when α(4.4.2.F) was expressed rather than α(2.2.2.F) (β2 P = 4 x 10^−6^ or β2(WLAGMI) P = 3 x 10^−4^). We conclude that potentiation conferred by alteration of the β2 subunit depends on the N-terminal region of the α subunit.

**Table 6:** Potentiation for α subunits expressed with wild-type β2 or β2 mutated to incorporate a region conferring potentiation by βEST.

Subunits	Potentiation ratio	ACh EC_50_ (μM)	Maximal response (-nA)
α(4.4.4.W)&β2	2.90 ± 0.27 (20) [0.12]	132 ± 10 (36)	13042 ± 1303 (51)
α(4.4.4.W)&β2(WLAGMI)	3.93 ± 0.53 (7)	121 ± 59 (2)	17069 ± 5490 (7)
α(2.2.4.W)&β2	1.45 ± 0.11 (5) [0.25]	248 ± 22 (4)	13033 ± 564 (11)
α(2.2.4.W)&β2(WLAGMI)	1.60 ± 0.06 (14)	717 ± 147 (4)	1397 ± 327 (14)
α(4.4.2.F)&β2	1.17 ± 0.03 (11) [<0.001]	81 ± 9 (4)	330 ± 132 (5)
α(4.4.2.F)&β2(WLAGMI)	1.38 ± 0.04 (16)	117 ± 10 (4)	10531 ± 1158 (16)
α(2.2.2.F)2&β2	0.96 ± 0.04 (12) [<0.001]	217 ± 42 (15)	130 ± 21 (26)
α(2.2.2.F)&β2(WLAGMI)	1.12 ± 0.03 (13)	619 ± 105 (3)	2354 ± 265 (13)

All α constructs were expressed at an 8:1 α:β ratio. The α subunits are named according to the subunit contributing the relevant region (see Text). Note that wild-type α4 is α(4.4.4.W) and wild-type α2 is α(2.2.2.F), but for ease of comparison all constructs are named in the same style. P values given are based on an unpaired t-test between the values for α subunits expressed with β2 or β2(WLAGMI).

## Discussion

Previous work had indicated that the final 4 residues of the α4 subunit (AGMI) are critical for potentiation, since removal or mutation of these residues reduced or removed potentiation by βEST. The tryptophan in the carboxy-terminal tail (WLAGMI) appeared to play a less important role [11]. The present results confirm the importance of the AGMI residues in binding βEST, while also demonstrating that the tryptophan is an important element in transduction of steroid binding to potentiation. Finally, the results demonstrate that the α4 subunit appears to be unique in having the ability to confer potentiation by βEST on heteromeric neuronal nicotinic receptors.

It was surprising that the α2 subunit did not allow potentiation given the overall sequence similarity between the two subunits (S2 Table) and the fact that in the C-terminal tail sequence the α2 subunit has the 4 residues previously identified as critical for potentiation (α2 FLAGMI and α4 WLAGMI). Previous work has examined the ability of βEST to potentiate heteropentameric receptors incorporating other α subunits. The α1 (the predicted C-terminal tail has sequence LIELNQQG) and α3 (LMAREDA) subunits do not support potentiation [9, 29]. The α5 subunit (IYKWANILIPVHIGNANK) does not produce functional receptors when it is the only α subunit in the pentamer, but when the α5 subunit was incorporated as a single copy into receptors in which the α4 subunit had been rendered non-competent it is not able to support potentiation by βEST [30]. The α6 subunit is difficult to express [31, 32], but given its sequence (LLGNTGKS) would appear unlikely to support potentiation.

The β subunit appears to play a secondary role in affecting potentiation. Wild-type β2 or β4 subunits do not confer potentiation when expressed with non-competent α subunits (α3: [9]; α2 present results) while receptors containing a competent α subunit and either β subunit can be potentiated, indicating that the nature of the β subunit is not decisive in permitting estrogen potentiation. It was previously noted that receptors containing the β2 subunit with the α4 subunit showed larger potentiation than when they contained the β4 subunit [9], and a similar trend was seen in the present study (7 of 9 α subunit constructs gave larger potentiation when expressed with the β2 than β4 subunit). We note that some of the regions found to remove potentiation from the α4 subunit (the A, D and E loops) are predicted to interact with an adjacent β subunit, perhaps providing a mechanism for this effect.

In contrast to our previous conclusions, the residue occupying the position of the W appears to strongly affect the efficacy of potentiation, indicating that it is involved in transduction between steroid binding and potentiation. The position of the W appears to be tightly constrained, in agreement with previous studies of the AGMI motif [11]. We examined the correlation between potentiation and a number of amino acid properties, but found no significant correlations and so the physical properties underlying this ability remain unknown. The properties included measures of the association of residues with the membrane interface, suggesting that anchoring of the TM4 helix is not the critical role.

Our results demonstrate that the extracellular amino-terminal portion of the α subunit also can affect the extent of potentiation by βEST. A possible role for the N-terminal portion of the α subunit had been suggested by previous work in which chimeras were made between the α3 and α4 subunits [9]. Those chimeras were constructed to transfer the entire region between the amino terminus and the start of the TM1 region, and it was found that the construct containing the N-terminal domain of the α3 subunit was potentiated to a significantly lesser extent than the full length α4 subunit. In our studies we could reduce potentiation by constructs based on the α4 subunit by inserting sequences from the amino-terminus of the α2 subunit, and conversely increase potentiation of constructs based on the α2 subunit by inserting homologous regions from the α4 subunit. The effective regions are predicted to be far from the membrane and the C-terminal tail. Furthermore, when we examined the ability of a β2 subunit mutated to confer potentiation (β2(WLAGMI)), the additional potentiation was larger when expressed with α subunits incorporating the α4 N-terminal region. These observations suggest that the effects of the N-terminal extracellular domain are not the result of close proximity of the N-terminal region to the C-terminal tail where they might contribute part of a binding site, or might involve direct interactions between residues in the C-terminal tail and the extracellular domain. Instead, they suggest that the amino-terminal region affects potentiation by an action on channel gating or on the transduction of steroid binding to potentiation. Our results also indicate that the contributions of the amino-terminal domain and the tryptophan residue are largely independent of each other.

We had previously hypothesized that the binding of 17β-estradiol altered the conformation of the membrane-spanning regions in such a way that stability of the open state of the channel was increased, resulting in potentiation [10]. This hypothesis was motivated by the finding that potentiation increased geometrically with the number of effective C-terminal regions present in the α4&β2 receptor, regardless of whether they were placed on α4 or β2 subunits. The present results on substitutions of the tryptophan residue suggest that the nature of this residue is important in generating the hypothesized conformational change.

Our studies of the role of the amino-terminal extracellular domain did not provide clear insights into the role of this region in potentiation. One possibility is that interactions between the N-terminal and transmembrane domains are altered. However, the residues proposed to be critical for this interaction are identical for α2 and α4, including E45 (numbering for the α1 subunit) and R209 [33] and F135 and R209 [34] in the N-terminal. The TM2-TM3 linker region has been proposed to form the portion of the transmembrane domain that interacts with residues in the N-terminal domain, but it is identical in α2 and α4 as well. Further, the mutations that removed potentiation from the α4 subunit are far from the membrane interface and located in the ACh-binding region of the receptor. Overall, the results of studies of the amino-terminal domain neither support nor contradict the hypothesis we advanced earlier regarding the mechanism for potentiation. On the one hand, they clearly indicate the importance of regions outside the C-terminal tail and the membrane-spanning helices in determining potentiation by βEST. On the other hand, they do not define an alternative physical mechanism.

There may not be a direct contradiction between a role of the extracellular domain and the proposed mechanism for potentiation based on altered conformation in the transmembrane regions. Allosteric models for channel gating imply that the energetics of channel opening and agonist binding are coupled: a receptor with an open channel also has higher affinity for agonist than a receptor with a closed channel. It is possible that in the α2 subunit some structural alterations in the N-terminal domain weaken a “reverse” coupling between the structural changes in the channel associated with potentiation and a change in the occupied ACh-binding site. An allosteric model such as this is also suggested by results obtained when the β2(WLAGMI) subunit is expressed with incompetent subunits (present results and [10]). These results indicate that the AGMI-binding and W-transduction motifs do not have to be on the α subunit, even though the N-terminal domains of the α subunit affect the magnitude of the resulting potentiation.

In sum, the results have indicated that potentiation of neuronal nicotinic receptors by βEST is a specific property of receptors containing the α4 subunit, and associated with the carboxyl-terminal tail of that subunit. Our previous studies found that the GMI motif is necessary for potentiation, as mutations to this region of the α4 subunit could completely remove potentiation [11] by reducing the apparent affinity of βEST. The tryptophan residue examined in the present study plays a significant role in transducing steroid binding to potentiation of the response to ACh. The residues in this region clearly are the dominant factor underlying the ability of estrogen potentiation. Our studies also identify a role for regions in the amino-terminal extracellular domain, albeit less significant, that modulate the efficacy of potentiation.

## Supporting Information

S1 Table Constructs used for regression of potentiation on activation. All α constructs were expressed with β4 at a 20:1 ratio. The data used for the regression included all constructs in Tables 2–5, with additional constructs. For convenience, all constructs are listed in S1 Table. The naming conventions are given in the appropriate Table legends. Regions in additional data are: FtoTM1 SGE(181) to V(210)IR; B to TM1 A(161)KID to V(210)IR; NtoA residue 1 to IVL(97); AtoTM1 N(58)AD to V(210)IR (numbers for mature α4). Point mutations in α2 are given the location in mature α2.
(DOCX)

S2 Table Amino acid identities to α4 for neuronal nicotinic α subunits. The first column identifies the neuronal nicotinic subunit, and the second gives the accession number for the sequence used. Mature subunit sequences were aligned (omitting the predicted signal sequence) using Clustal Omega (http://www.ebi.ac.uk/Tools/msa/clustalo/; McWilliam et al. Nucleic Acids Research 2013 41: W597–600 10.1093/nar/gkt376). The number of amino acids in the regions specified that were identical to the aligned position in the α4 subunit were determined and the fraction of identical residues calculated as the fraction of residues in α4. The regions used were (residues numbered as in mature α4): N-terminal 1–210; TM1-TM3: 211–300; Cytoplasmic loop: 301–570; TM4 to C-terminal: 571–598.
(DOCX)
