# Supplementary material for: Potentiation of Neuronal Nicotinic Receptors by 17β-Estradiol: Roles of the Carboxy-Terminal and the Amino-Terminal Extracellular Domains
Source: PLoS One. 2015 Dec 18;10(12):e0144631. doi: 10.1371/journal.pone.0144631 (PMC4684330; doi:10.1371/journal.pone.0144631)
Supplement: S1 Table — All α constructs were expressed with β4 at a 20:1 ratio. The data used for the regression included all constructs in Tables 2–5, with additional constructs. For convenience, all constructs are listed in S1 Table. The naming conventions are given in the appropriate Table legends. Regions in additional data are: FtoTM1 SGE(181) to V(210)IR; B to TM1 A(161)KID to V(210)IR; NtoA residue 1 to IVL(97); AtoTM1 N(58)AD to V(210)IR (numbers for mature α4). Point mutations in α2 are given the location in mature α2. (DOCX) [file pone.0144631.s001.docx]

**Table S1 Constructs used for regression of potentiation on activation.**

| **Source** | **Potentiation ratio** | **ACh EC_50_ (μM)** | **Maximal response (-nA)** |
| --- | --- | --- | --- |
| **Table 2** |  |  |  |
| **α2 wt** α(2.2.2.F) | 1.01 ± 0.02 (30) | 91 ± 6 (28) | 929 ± 102 (85) |
| α(2.2.4.F) | 0.97 ± 0.02 (16) | ND | 2526 ± 567 (17) |
| α(2.4.4.F) | 1.02 ± 0.02 (8) | ND | 3367 ± 627 (20) |
| α(4.4.2.F) | 1.15 ± 0.01 (20) | 46 ± 10 (3) | 7827 ± 615 (28) |
| α(4.4.4.F) | 1.25 ± 0.02 (35) | 32 ± 3 (3) | 9146 ± 505 (42) |
| α(2.2.2.W) | 1.31 ± 0.03 (31) | 103 ± 3 (9) | 751 ± 66 (38) |
| α(4.2.2.W) | 1.43 ± 0.03 (14) | ND | 2083 ± 314 (12) |
| α(2.4.4.W) | 1.54 ± 0.04 (23) | 76 ± 3 (3) | 6881 ± 602 (23) |
| α(2.2.4.W) | 1.54 ± 0.04 (10) | 99 ± 6 (6) | 651 ± 85 (20) |
| α(4.4.2.W) | 1.66 ± 0.07 (12) | 41 ± 5 (2) | 9039 ± 673 (12) |
| **α4 wt** α(4.4.4.W) | 1.87 ± 0.04 (80) | 31 ± 2 (21) | 10607 ± 473 (118) |
| **Table 3** |  |  |  |
| α4(D) | 1.04 ± 0.01 (12) | 22 ± 2 (5) | 10398 ± 1333 (13) |
| α4(Y) | 1.33 ± 0.03 (12) | ND | 2394 ± 382 (12) |
| α4(G) | 1.46 ± 0.04 (15) | ND | 3001 ± 503 (17) |
| α4(L) | 1.77 ± 0.18 (7) | ND | 7418 ± 632 (7) |
| α4(A) | 1.88 ± 0.03 (4) | ND | 7390 ± 719 (4) |
| α4(S) | 1.90 ± 0.05 (11) | 21 ± 2 (6) | 10964 ± 927 (11) |
| α4(K) | 1.97 ± 0.14 (10) | 14 ± 2 (7) | 12244 ± 1392 (11) |
| **Table 4** |  |  |  |
| α4(PPFLAGMI) | 1.25 ± 0.02 (35) | 32 ± 3 (3) | 9146 ± 505 (42) |
| α4(WPFLAGMI) | 1.32 ± 0.06 (5) | 7 ± 2 (4) | 11522 ± 1941 (5) |
| α4(PWFLAGMI) | 1.05 ± 0.05 (8) | 7 ± 2 (8) | 15190 ± 1725 (10) |
| α4(PPFWAGMI) | 1.36 ± 0.06 (6) | 14 ± 1 (4) | 12886 ± 1447 (8) |
| **Table 5** |  |  |  |
| α4(α2DtoA) | 1.70 ± 0.06 (4) | 76 ± 12 (4) | 12160 ± 390 (11) |
| α4(α2E) | 1.83 ± 0.09 (14) | 6.9 ± 0.5 (4) | 7765 ± 366 (4) |
| α4(α2DtoA+E) | 1.59 ± 0.04 (5) | ND | 13221 ± 926 (12) |
| α4(F)(α2DtoA) | 1.27 ± 0.01 (13) | 73 ± 4 (4) | 13760 ± 1143 (13) |
| α4(F)(α2Eloop | 1.16 ± 0.02 (15) | 8.5 ± 0.6 (11) | 7173 ± 705 (15) |
| α4(F)(α2DtoA+E) | 0.97 ± 0.03 (13) | 16 ± 2 (6) | 12361 ± 1017 (14) |
| α2(W)(α4DtoA) | 1.35 ± 0.04 (15) | 77 ± 7 (7) | 211 ± 30 (15) |
| α2(W)(α4DtoA+E) | 1.50± 0.04 (17**)** | 318± 30 (7) | 145± 16 (17) |
| α2(α4DtoA+E) | 1.04 ± 0.01 (13) | 257 ± 12 (23) | 544 ± 42 (39) |
| **Additional data** |  |  |  |
| α2(α4FtoTM1) | 0.95 ± 0.06 (5) | 37 ± 2 (9) | 376 ± 81 (42) |
| α2(α4NtoA) | 1.08 ± 0.09 (6) | 96 ± 3 (7) | 1607 ± 990 (15) |
| α2(α4E+BtoTM1) | 1.04 ± 0.01 (10) | 159 ± 6 (5) | 520 ± 62 (32) |
| α2(α4AtoTM1) | 1.06 ± 0.01 (13) | 211 ± 32 (7) | 2169 ± 321 (24) |
| a(α2NtoA) | 1.84 ± 0.03 (5) | ND | 9720 ± 1300 (14) |
| a4(PPWAGM) | 0.95 ± 0.01 (4) | ND | 11733 ± 820 (4) |
| a4(PLWLAGMI) | 1.50 ± 0.18 (7) | 15 ± 5 (4) | 12677 ± 1009 (7) |
| α2(L465M) | 1.00 ± 0.01 (9) | ND | 732 ± 144 (9) |
| α2(F471L) | 1.05 ± 0.02 (25) | 117 ± 5 (5) | 725 ± 80 (27) |
| α2(I475V) | 1.03 ± 0.01 (24) | ND | 291 ± 40 (29) |

All α constructs were expressed with β4 at a 20:1 ratio. The data used for the regression included all constructs in Tables 2 to 5, with additional constructs. For convenience, all constructs are listed in Table S1. The naming conventions are given in the appropriate Table legends. Regions in additional data are: FtoTM1 SGE(181) to V(210)IR; B to TM1 A(161)KID to V(210)IR; NtoA residue 1 to IVL(97); AtoTM1 N(58)AD to V(210)IR (numbers for mature α4). Point mutations in α2 are given the location in mature α2.
