# Supplementary material for: Potentiation of Neuronal Nicotinic Receptors by 17β-Estradiol: Roles of the Carboxy-Terminal and the Amino-Terminal Extracellular Domains
Source: PLoS One. 2015 Dec 18;10(12):e0144631. doi: 10.1371/journal.pone.0144631 (PMC4684330; doi:10.1371/journal.pone.0144631)
Supplement: S2 Table — The first column identifies the neuronal nicotinic subunit, and the second gives the accession number for the sequence used. Mature subunit sequences were aligned (omitting the predicted signal sequence) using Clustal Omega (http://www.ebi.ac.uk/Tools/msa/clustalo/; McWilliam et al. Nucleic Acids Research 2013 41: W597–600 10.1093/nar/gkt376). The number of amino acids in the regions specified that were identical to the aligned position in the α4 subunit were determined and the fraction of identical residues calculated as the fraction of residues in α4. The regions used were (residues numbered as in mature α4): N-terminal 1–210; TM1-TM3: 211–300; Cytoplasmic loop: 301–570; TM4 to C-terminal: 571–598. (DOCX) [file pone.0144631.s002.docx]

**Table S2. Amino acid identities to α4 for neuronal nicotinic α subunits**

| subunit | Accession | N-terminal | TM1-TM3 | Cytoplasmic loop | TM4 to C-terminal |
| --- | --- | --- | --- | --- | --- |
| α2 | NP_659052.1 | 0.77 | 1.00 | 0.29 | 0.86 |
| α3 | NP_000734.2 | 0.60 | 0.94 | 0.27 | 0.62 |
| α4 | NP_000735.1 |  |  |  |  |
| α5 | NP_000736.2 | 0.57 | 0.76 | 0.24 | 0.41 |
| α6 | NP_004189.1 | 0.60 | 0.89 | 0.28 | 0.62 |

The first column identifies the neuronal nicotinic subunit, and the second gives the accession number for the sequence used. Mature subunit sequences were aligned (omitting the predicted signal sequence) using Clustal Omega (<http://www.ebi.ac.uk/Tools/msa/clustalo/>; McWilliam et al. Nucleic Acids Research 2013 41: W597-600 doi:10.1093/nar/gkt376). The number of amino acids in the regions specified that were identical to the aligned position in the α4 subunit were determined and the fraction of identical residues calculated as the fraction of residues in α4. The regions used were (residues numbered as in mature α4): N-terminal 1 - 210; TM1-TM3: 211-300; Cytoplasmic loop: 301-570; TM4 to C-terminal: 571-598.
